# The Physiological Anatomy and Physiology of Man

**Published:** 1843-04

**Authors:** 


ART. III.—*The Physiological Anatomy and Physiology of Man.* By ROBERT B. TODD, M.D. F.R.S., Professor of Physiology in King's College, London; and WILLIAM BOWMAN, F.R.S., Demonstrator of Anatomy in King's College, London. Part I. with Fifty-one Wood Engravings.—*London*, 1843. 8vo, pp. 200.

THE plan of the present treatise is in many respects new; and we believe it to be admirably adapted to meet an existing want. Its design is, to afford to the student and practitioner a plain and accurate view of the intimate structure and functions of the human body; and its arrangement corresponds with that which is adopted in the physiological course at King's College. All the subjects have been wrought out anew by the authors conjointly, and the work is the product of their united labours. The reputation of Dr. Todd as an able and successful teacher of physiology, and that of Mr. Bowman as an accomplished microscopist, afford a guarantee that their projected treatise will be executed in a manner worthy of the subject; and our examination of the portion already published has fully satisfied us of its value. We are not certain, however, that it can be rightly called a treatise on physiology; since, in that science, the *function* and its conditions are the objects of inquiry, and the *organ* is considered merely as subservient to it. Thus in discussing the respiratory function, the physiologist has to include the skin as well as the lungs, and must also explain the mechanism by which the respiratory movements are effected. In the work before us, the *organology* is the prominent part, and the physiology is generally made subservient to it. Hence we think the first part of the title more appropriate than the second; and as a Treatise on Physiological Anatomy,—not superseding the various systems of physiology at present in use, but to be read in conjunction with them or rather before them, we strongly recommend this work to the attention of our readers. It seems to us to contain exactly what the student ought to know on every subject discussed in it; and the descriptions are so clear, and the illustrations so complete, as to leave nothing to wish for. This first part contains, after an introductory chapter on Life and Organization, a general account of the solid and fluid constituents of the body, a description of the various minute movements occurring in its interior, and a detailed examination of the tissues classed under the head of "passive and active organs of locomotion," including the fibrous, adipose, areolar, cartilaginous, osseous, serous, and muscular structures. We prefer reserving a more detailed analysis, until the whole shall have been completed.

